# Tissue Injury Protection: The Other Face of Anticoagulant Treatments in the Context of Ischemia and Reperfusion Injury with a Focus on Transplantation

**DOI:** 10.3390/ijms242417491

**Published:** 2023-12-14

**Authors:** Julie Carré, Thomas Kerforne, Thierry Hauet, Laurent Macchi

**Affiliations:** 1 Service D’Hématologie Biologique, Centre Hospitalo-Universitaire de Poitiers, 86000 Poitiers, France; laurent.macchi@univ-poitiers.fr; 2 INSERM 1313 Ischémie Reperfusion, Métabolisme, Inflammation Stérile en Transplantation (IRMETIST), Université de Poitiers, 86000 Poitiers, France; thomas.kerforne@chu-poitiers.fr (T.K.); thierry.hauet@inserm.fr (T.H.); 3 Service D’Anesthésie-Réanimation et Médecine Péri-Opératoire, Centre Hospitalo-Universitaire de Poitiers, 86000 Poitiers, France; 4 FHU Survival Optimization in Organ Transplantation (SUPORT), 86000 Poitiers, France; 5 Service de Biochimie, Centre Hospitalo-Universitaire de Poitiers, 86000 Poitiers, France

**Keywords:** anticoagulant, ischemia–reperfusion injury, thrombo-inflammation, transplantation, coagulation

## Abstract

Organ transplantation has enhanced the length and quality of life of patients suffering from life-threatening organ failure. Donors deceased after brain death (DBDDs) have been a primary source of organs for transplantation for a long time, but the need to find new strategies to face organ shortages has led to the broadening of the criteria for selecting DBDDs and advancing utilization of donors deceased after circulatory death. These new sources of organs come with an elevated risk of procuring organs of suboptimal quality. Whatever the source of organs for transplant, one constant issue is the occurrence of ischemia–reperfusion (IR) injury. The latter results from the variation of oxygen supply during the sequence of ischemia and reperfusion, from organ procurement to the restoration of blood circulation, triggering many deleterious interdependent processes involving biochemical, immune, vascular and coagulation systems. In this review, we focus on the roles of thrombo-inflammation and coagulation as part of IR injury, and we give an overview of the state of the art and perspectives on anticoagulant therapies in the field of transplantation, discussing benefits and risks and proposing a strategic guide to their use during transplantation procedures.

## 1. Introduction

Organ transplantation stands as one of modern medicine’s most remarkable achievements, offering a lifeline to countless individuals facing life-threatening organ failures. Over the past few decades, this medical achievement has evolved from a pioneering experiment into a mainstream therapeutic solution, dramatically enhancing the quality of life and extending the survival of patients suffering from end-stage organ diseases [1,2,3,4,5]. Despite its remarkable success, organ transplantation confronts an enduring and vexing challenge: the persistent scarcity of viable organs. In numerous countries, the primary source of organs for transplantation has traditionally been donors deceased after brain death (DBDDs) [6]. However, over the last two decades, in response to the ongoing challenge of organ shortage, various strategies have been explored: broadening the criteria for selecting DBDDs [7,8,9], advancing utilization of donors deceased after circulatory death (DCDDs) [10] and a minority use of living donors. Organs procured from living donors often exhibit exceptional quality, but their applicability is limited to a select range of organs, and their contribution to the overall organ pool remains relatively modest.

Thus, the most pivotal solution to organ shortage continues to be the expansion of the deceased donor pool. This expansion, however, comes with inherent challenges. As the pool of eligible donors widens, there is an elevated risk of procuring organs of suboptimal quality or those that are not usable [7,8,10,11]. This, in turn, can manifest in issues such as delayed graft function, early graft failure and abbreviated graft survival. These observations find potential elucidation in the escalating susceptibility of these organs to ischemia–reperfusion (IR) injury, giving rise to a cascade of events, including the activation of coagulation and inflammation pathways. This activation subsequently culminates in tissue lesions (which generate damage-associated molecular patterns, or DAMPs), endothelial activation and the initiation of innate immune responses characterized by the involvement of the complement system and cellular reactions. Additionally, this process prompts the infiltration of tissues by macrophages, further exacerbating the damage. As a direct consequence of these tissue impairments, occurrences such as the no-reflow phenomenon, delayed graft function and an increased likelihood of graft loss emerge. This interplay underscores the pivotal role that IR injury and coagulation/inflammation pathways play in shaping the fate of these organs post-transplantation, necessitating comprehensive strategies to mitigate their impact and enhance the overall success of graft outcomes.

## 2. Thrombo-Inflammation in Ischemia–Reperfusion Injuries

### 2.1. Ischemia Reperfusion Injury

Several clinical situations may lead to ischemia and reperfusion of organs, such as arterial occlusion, circulatory arrest, transplantation, etc., in which O_2_ supply is temporarily interrupted before blood circulation, and therefore oxygenation, is restored. This sequence of ischemia and reperfusion may be responsible for tissue injuries and organ failure. IR injury has been described in various organs, such as the brain; after cardiac arrest [12]; in myocardial infarction [13]; in kidney transplantation, inducing delayed graft function [14]; or in liver transplantation, with a greater impact caused by steatosis [15], etc. It appears that, while reperfusion is essential for the recovery of organ function, it also participates in and aggravates IR injury by itself [13]. Moreover, multiorgan dysfunction is likely to occur after IR injury of an isolated organ [16].

IR injury results from metabolic and cellular disorders caused by hypoxia and abrupt reoxygenation. Combined, these disorders contribute to a deleterious state called thrombo-inflammation. In contrast with thrombo-inflammation caused by sepsis, thrombo-inflammation in IR takes place in a sterile environment but still results in immune response, coagulation trigger, cellular activation and sometimes cell death [14,17].

### 2.2. Thrombo-Inflammation: Central Role of Endothelial Cells

Thrombo-inflammation is an exaggerated response to various cellular stresses. Its process can be described as interdependence between thrombosis and inflammation involving circulating blood cells, the coagulation system, and innate and adaptive immunity, as well as endothelial cells. These endothelial cells occupy a key place in thrombo-inflammation, as they interact with many other cells via coagulation and inflammation mediators, thus making microcirculation the main site of thrombo-inflammation [18,19].

**Figure 1:**
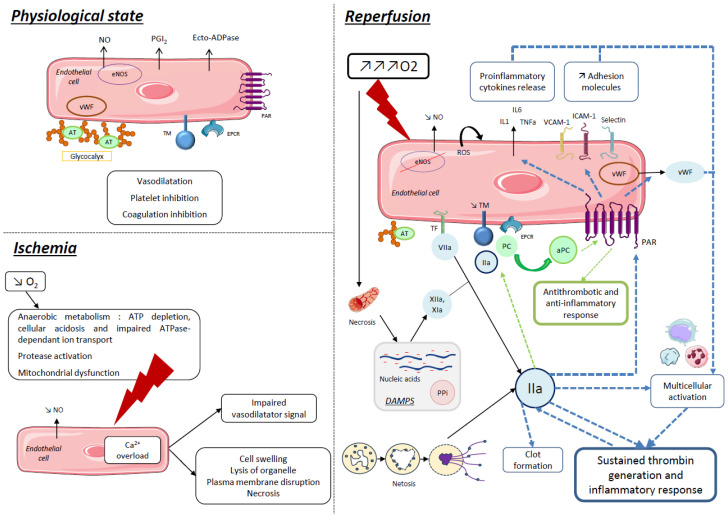
Cellular and hemostatic approaches to physiopathological mechanisms in ischemia–reperfusion injury. In a physiological state, endothelial cell displays a thromboresistant phenotype with antiplatelet (PGI_2_, NO, Ecto-ADPase, etc.), anticoagulant (TM, EPCR, AT, etc.) and anti-inflammatory effects (TM, EPCR, PAR, etc.). During ischemia–reperfusion, hypoxia and reoxygenation lead to abnormal oxidative metabolism and oxidative stress, to the loss of endothelial thromboresistant and vasodilatating properties and to endothelial barrier disruption. During reperfusion, oxidative stress is amplified and endothelial cells are a source of thrombo-inflammation because of pro-inflammatory cytokine release, adhesion molecule expression, TF expression and decreased AT and TM expression leading to sustained thrombin generation and inflammatory response, as well as decreased antithrombotic and anti-inflammatory response [19]. aPC: activated protein C; AT: antithrombin; ATP: adenosine triphosphate; eNOS: endothelial nitric oxide synthase; DAMPs: damage-associated molecular patterns; EPCR: endothelial protein C receptor; ICAM-1: intracellular adhesion molecule; IL: interleukin; NO: nitric oxide; PAR: protease activated receptor; PC: protein C; PGI_2_: prostacyclin; PPi: inorganic polyphosphates; ROS: reactive oxygen species; TM: thrombomodulin; TNF: tumor necrosis factor; VCAM-1: vascular cell adhesion molecule; vWF: von Willebrand factor.

Resting endothelial cells usually display antithrombotic and anti-inflammatory properties, thanks to the molecules they express, release in their environment or contain in the glycocalyx, such as nitric oxide (NO), prostacyclin (PGI_2_), ecto-ADPase and anticoagulant proteins [20] (Figure 1).

NO is produced thanks to endothelial NO synthase (eNOS) and released by endothelial cells in their environment. In vascular physiology, NO is involved in a variety of functions (regulation of vascular tone, maintenance of vascular permeability, inhibition of smooth muscle cells, stimulation of endothelial cell regeneration), immune mechanisms and interactions with blood cells in limiting their activation (inhibition of platelet activation and leukocyte adherence to endothelium) [21]. 

Under pathological conditions such as ischemia–reperfusion, sepsis or disseminated intravascular coagulation, thrombo-inflammation leads to disruption of endothelial cell properties and thus to endothelial dysfunction. 

IR injury results in sterile inflammation and has been described as a cause of transplantation failure [22,23]. Indeed, oxidative disorders caused by insufficient O_2_ supply and then abrupt O_2_ inflow are responsible for increasing reactive oxygen species (ROS) production, eNOS deregulation with a decrease in endothelial NO production and cell death. Besides the intrinsic toxicity of nucleic acids, proteins and lipids degradation, ROS accumulation participates in NO decrease and so worsens the loss of vascular integrity and endothelial thromboresistance. NO decrease and ROS accumulation both provoke NFκB activation-enhancing synthesis of inflammatory proteins by endothelial cells and leukocytes, such as tumor necrosis factor (TNF) α and β, interleukins (IL1b, IL6, etc.), chemokines (IL8, MIP-1, MCP-1, etc.) and adhesion molecules (ICAM-1, VCAM-1, P-selectin, E-selectin) (Figure 1). As a result, endothelial dysfunction triggers leukocyte recruitment and extravasation and platelet recruitment and activation [18].

In this review, we focus on the coagulation side of thrombo-inflammation, and we present the state of the art of anticoagulant therapies currently used and the potential progress to be expected from these drug families to come.

### 2.3. Modulation of Thrombo-Inflammation by Coagulation Serine Proteases and Protease-Activated Receptors

#### 2.3.1. Procoagulant Factors

Coagulation activation and thrombin generation is recognized as an important feature of thrombo-inflammation [19]. It is accepted by consensus that the coagulation process is mostly initiated via the tissue factor (TF) pathway, when TF is exposed via the subendothelium after a vessel injury and forms a complex with activated factor VII (FVIIa). In IR injury, TF is expressed by monocytes, endothelial cells and circulating microparticles, resulting in thrombin generation. However, it seems that in IR injury, the contact pathway of coagulation is also significantly involved, thanks to the effects of negatively charged molecules released by damaged or dying cells (nucleic acids, inorganic polyphosphates, etc.) [24,25]. Both of these pathways lead to factor X (FX) activation and thrombin generation, which is self-sustained via the feedback activation of factor XI (FXI) and other factors and via a multicellular activation favoring a procoagulant and pro-inflammatory phenotype (endothelial cells, platelets, monocytes, neutrophils, etc.) [26]. In addition, von Willebrand factor (vWF) is released by activated endothelial cells and contributes to platelet activation, thus enhancing thrombin generation [27].

Thrombin generation is also promoted by neutrophil extracellular traps (NETs) made of DNA–histone complexes and proteins released from activated neutrophils in the context of inflammation or hypoxia, forming a network which is likely to activate a coagulation cascade thanks to the negative charges of nucleic acids and abundant enzymes. In pathological conditions, these NET byproducts are produced in exaggerated or uncontrolled amounts and may also interfere with physiologic coagulation inhibitor’s activity (see below) and activate endothelial cells and platelets [28,29].

#### 2.3.2. Anticoagulant Factors

Antithrombin (AT), protein C (PC), protein S (PS) and tissue factor pathway inhibitor (TFPI) are the main physiological coagulation inhibitors. They target procoagulant factors to prevent excessive coagulation. Deficiency in one of them may result in a predisposition towards venous thrombo-embolism [30]. AT is a strong inhibitor of thrombin and FXa and, to a lesser extent, of some other procoagulant factors. It has the ability to bind to glycosaminoglycans forming the glycocalyx and participates in the anticoagulant capacity of the endothelial layer [31]. TFPI is another coagulation inhibitor localized in the glycocalyx [24]. However, as glycocalyx integrity is altered with IR, and specifically in transplantation, the endothelium consequently loses these glycocalyx-related anticoagulant properties, develops a procoagulant phenotype and is directly exposed to inflammatory and oxidative molecules [32,33,34]. PC is part of a system called the protein C system. It is composed of PC, thrombin, thrombomodulin (TM) and endothelial protein C receptor (EPCR), which are all transmembrane proteins on the endothelial cell membrane. This whole complex is required to turn the PC into an activated PC (aPC), finally associated with its cofactor PS. Together, they inhibit activated factor V (FVa) and activated factor VIII (FVIIIa), thus limiting excessive coagulation and trapping thrombin in the anticoagulant pathway [35]. In hypoxic or inflammatory conditions, it has been shown that TM expression is decreased in endothelial cells in vitro [36,37], and that this decrease contributes to IR injury in vivo [38].

#### 2.3.3. Protease-Activated Receptors

Another mechanism of action of thrombin requires the activation of protease-activated receptors (PAR-1 to -4), which belong to the G protein-coupled receptor (GPCR) family and are expressed on several cellular types such as endothelial cells, platelets, etc. They are activated by proteolytic cleavage. Indeed, the PAR extracellular amino-terminus domain is cleaved by a protease, exposing a new amino-terminus binding to the receptor itself and called a tethered ligand [39]. These are biased receptors, meaning that they trigger different cellular effects depending on the activator involved. Indeed, proteases that activate PAR have different cleavage sites, thus triggering different PAR intracellular signals. In this way, when activated by thrombin, PAR-1 has procoagulant and pro-inflammatory effects, while when activated by aPC it has anti-inflammatory and cytoprotective effects [40].

### 2.4. Ischemia–Reperfusion Injury and Thrombo-Inflammation in the Field of Transplantation

Different strategies have been approved to limit IR injury and thrombo-inflammation inherent to transplantation procedure.

In both kidney and liver transplants, organ-protective treatments are supplied to the recipient in addition to anesthetic, immunosuppressive and antibiotic medication. Indeed, they receive pharmacological molecules to control blood pressure and blood flow, mainly crystalloids, diuretics and/or vaso-active drugs. Although thrombosis is not uncommon, heparin administration remains unusual given the major hemorrhagic risk and depends on the transplanted organ. In pancreatic transplantation, heparin may even be associated with antiplatelet agents [41,42].

Preservative strategies for organs before transplantation are also considered a key driver of reducing IR injury during transplantation. Barba et al. showed that increase in the duration of cold ischemia increased the risk of transplant failure [22]. Initially, the gold standard was static–cold organ preservation. Evolution of techniques made possible the development of ex vivo machine perfusion. Studies are still ongoing to determine and/or improve the optimal perfusion solution and its gaseous composition, system temperature, flow conditions, etc. Currently, perfusate contains several pharmacologic components to maintain vascular homeostasis and is heparinized to avoid thrombosis of the system [43].

As IR is responsible for thrombo-inflammation, which contributes to organ failure, coagulation pathways could be a proper target before, during or after the procedure to improve transplantation outcomes via decrease of thrombo-inflammation.

In a rat model of IR injury via partial aortic clamp, Sharfuddin et al. observed a rapid alteration of kidney function and structure, with a diminution of blood flow and loss of vascular integrity until 4 days after reperfusion [44]. These observations are consistent with the microvascular dysfunction described during the “no reflow” phenomenon, where edema due to increase of endothelial permeability, leukocytes adherence and imbalance between vasodilatory and vasoconstrictive mediators prevent effective blood flow recovery in the reperfusion stage, thus exacerbating IR injury [45]. It is now clear that endothelium, via endothelial dysfunction, plays a major role in IR injury by promoting inflammation and thrombin generation (Figure 1). Thus, as inflammation and coagulation are linked via thrombo-inflammation, it is legitimate to bet on inhibiting the coagulation pathway to limit thrombo-inflammation and IR injury. To this end, Sharfuddin et al. administrated soluble TM as a pretreatment before partial aortic clamp in rats and observed improvement of kidney function, reduction in the severity of renal injury, increase in blood flow and preservation of vascular integrity in treated rats compared to controls. They even showed that treatment only 2 h after reperfusion may decrease mortality in treated rats [44].

These results confirm that inhibiting procoagulant pathways may be of interest in reducing ischemia–reperfusion injury.

In the case of DBDDs, blood flow is maintained until organ harvesting. Then, preservation consists of cold or warm ischemia. Finally, the harvested organ undergoes a final stage of warm ischemia before reperfusion. In the case of DCDDs, blood flow is interrupted before organ harvesting, which is responsible for prolonged warm ischemia before organ retrieval. These different situations must be kept in mind when determining the optimal time for treatment administration. moreover, the molecule and the way it is administered remain to be studied.

## 3. Anticoagulant Therapies and Ischemia–Reperfusion Injuries

Interplay between inflammation and thrombin inhibition have suggested that anticoagulant (AC) therapy could be a suitable means of modulating thrombo-inflammation and ameliorated IR-induced tissue injuries. When considering AC therapies, these could be divided into direct ACs and indirect ACs (vitamin K antagonists, VKA, are beyond the scope of this review considering their length of action, which is not suitable to these clinical conditions). Indirect ACs include unfractionated heparin (UFH), low molecular-weight heparin (LMWH) and fondaparinux. Direct ACs can be divided into direct anti-Xa (rivaroxaban, apixaban, edoxaban) and anti-IIa molecules (dabigatran, argatroban, bivalirudin). These therapies, by inhibiting one or several serine proteases, could be monitored to evaluate their impact on coagulation cascade. In clinical practice, global and specific tests are used. Regarding UFH therapy, activated partial thromboplastin time (APTT) is widely used despite some pitfalls, and specific tests such anti-Xa activity evaluation are preferred [46]. LMWH, fondaparinux and oral direct ACs are monitored via anti-Xa assays with specific calibrations for each of these molecules. With these tests we are able to “quantify” the anticoagulant effect of these drugs [46,47]. Another approach includes global tests such as thrombo-elastography and thrombin generation testing (TGT), which evaluate hemostasis status and/or the capacity of a patient’s plasma to generate thrombin. However, these global tests, particularly thrombo-elastography, are not designed to monitor anticoagulant treatments specifically, despite their particular interest regarding liver transplantation and cardiac surgery [48].

### 3.1. Indirect Anticoagulant Therapies and Tissue Injury

Indirect AC therapy protection, which includes UFH, LMWH and fondaparinux, needs AT to exert its anticoagulant properties. These three “classes” of molecules exert their activities in differently inhibiting the serine protease thrombin and FXa. UFH inhibits thrombin and FXa equally, and LMWH preferentially inhibits FXa, while fondaparinux has an exclusive anti-FXa activity. Due to their different mode of inhibition of serine proteases, their effect on the improvement of IR lesions may, for example, make it possible to better understand their mechanisms of action independently of their anticoagulant effect via PAR.

#### 3.1.1. Unfractionated Heparin

UFH has been extensively studied in different IR models (Table 1). In an ex vivo isolated perfused heart model, administration of heparin before ischemia improved myocardial function [49]. This effect was independent of the AC effect of heparin, since the same beneficial effect was obtained with N-acetyl-heparin devoid of anticoagulant action. In a canine model of myocardial IR, UFH administered via three intravenous (IV) injections (one during ischemia and two at the start of reperfusion) decreased the final infarct size, again independently of the AC effect of the molecule [50]. It is noticeable that in these various in vivo studies, the dose of UFH used was 2 to 6 times higher than the dose used in human therapy, without hemorrhagic consequences. A study using lower doses found no cardioprotective effect from UFH in dogs [51]. The choice of UFH dosage seemed to be very important. Kouretas et al. found that UFH, in a myocardial IR model, improved myocardial contractility by preserving the vasodilator functions of the coronary endothelium via the synthesis of NO [52]. Other IR models have demonstrated beneficial effects from UFH, including a hepatic IR models in pigs [53] and rabbits [54], a mesenteric IR model in rats [55] and a model of cerebral IR in rats [56]. Among the mechanisms underlying this protective effect, a decrease in leukocyte infiltration is frequently mentioned [56]. Thus, UFH appears to be protective in IR injury independent, of its anticoagulant effect, at doses higher than those used for antithrombotic purposes in humans and related to anti-inflammatory properties.

#### 3.1.2. Low Molecular-Weight Heparin

LMWH has been studied in a variety of IR models (Table 1). In myocardial infarction models, enoxaparin uniformly shows a protective effect when administered before reperfusion [51,57,58] via its anti-inflammatory effect and/or the decrease of neutrophil accumulation. Other models of IR have demonstrated beneficial effects on IR injuries, including in mesenteric, liver, cerebral and kidney models [59,60,61,62,63].

#### 3.1.3. Fondaparinux

Fondaparinux, a synthetic pentasaccharide, is an LMWH analogue that selectively and indirectly inhibits FXa by binding to AT. This molecule has demonstrated protective effects against IR in different models (Table 1). In a mouse model of renal IR, fondaparinux has been shown to decrease fibrin formation, inflammation and neutrophil accumulation in the kidneys [64]. In the same model, this team used modified fondaparinux, which had lost its affinity for AT and therefore its AC action. This modified pentasaccharide has been shown to be as effective as fondaparinux in terms of cardioprotection as well as in inhibiting cell adhesion to P-selectin, thus demonstrating a cardioprotective and anti-inflammatory effect independent of the anticoagulant effects [86]. In a model of intestinal IR in rats, Olanders’s team did not find any beneficial effect of fondaparinux on endothelial permeability or on leukocyte infiltration, despite a decrease in the plasma concentration of the cytokine pro-inflammatory macrophage inflammatory protein 2 (MIP-2) [65]. This lack of anti-inflammatory effect could be explained by an insufficient dose, fondaparinux having been administered subcutaneously in this study. Intraperitoneal or IV administration is more commonly used in studies conducted on fondaparinux in IR, with circulating concentrations 5 to 10 times higher than those used in humans [64,67]. The anti-inflammatory properties of fondaparinux in myocardial IR were studied in an isolated heart model, showing a beneficial effect of fondaparinux on myocardial function and decreased leukocyte infiltration [66]. In an in vivo myocardial IR model, we found that fondaparinux displayed a cardioprotective effect via a decrease in infarct size [67]. This cardioprotective effect has been shown to be related to the phosphorylation of the transcription factor STAT3 and upregulation of TM and EPCR on endothelial cells without anti-inflammatory effect [87]. In the context of renal transplantation, fondaparinux would be of interest in terms of organ preservation, since the administration of this molecule in a porcine model of renal autotransplantation led to an improvement in renal function and a reduction in fibrosis and leukocyte infiltration [68]. It is of interest to note that in the treated group, cleaved PAR-2 receptors (activated receptor) were lower than in the control group [68], suggesting that anti-FXa drugs could be protective in limiting PAR activation.

### 3.2. Direct Anticoagulant Therapies and Tissue Injury

#### 3.2.1. Xabans (Rivaroxaban, Apixaban)

Rivaroxaban and apixaban, which directly inhibit FXa, have been studied in IR models. Several studies have focused on a model of cerebral ischemia in rats, regarding reversible ligation of the middle cerebral artery [70,71,72,88]. Among these various studies, some of them focused on the risk of hemorrhagic transformation linked to thrombolysis performed concurrently with anticoagulation via rivaroxaban [70,72,88]. Overall, these studies showed a reduction in the risk of intracerebral hemorrhage with rivaroxaban via a protective effect on the neurovascular unit [88], made up of endothelial cells, astrocytes and pericytes [70,72]. Interestingly, in one of these studies, rivaroxaban was shown to cause a decrease in the expression of PAR-1 and PAR-2 in the brain compared to warfarin [72]. The only team that demonstrated a neuroprotective effect with reduction in final infarct size with rivaroxaban was that of Dittmeier et al. [71], who demonstrated an anti-inflammatory effect with a reduction in macrophage infiltrate as well as a reduction in the expression of ICAM-1 in the brain. In other studies, neither rivaroxaban nor apixaban reduced infarct size [70,72]. In a model of mesenteric vein thrombosis in diabetic mice, rivaroxaban caused a decrease in leukocyte adhesion, suggesting an anti-inflammatory effect [69]. In a model of coronary ligation followed by reperfusion, we demonstrated that therapeutic dosage of rivaroxaban decreases infarct size in rats [73]. In this study, rivaroxaban exerted a cytoprotective effect on H9c2 cells (cardiomyoblasts) submitted to hypoxia/reoxygenation.

#### 3.2.2. Direct Thrombin Inhibitors

1. Hirudin

Direct inhibition of thrombin with hirudin reduced myocardial reperfusion injury in rabbits [74]. This effect was independent of thrombin-triggered fibrin formation, since administration of a fibrinogen-depleting agent in the same model did not modify the final infarct size. In this study, the protective effect of hirudin was linked to the inhibition of the expression of IL-8 and MCP-1 as well as to a significant reduction in leukocyte infiltration in the myocardial risk area. The beneficial effects of hirudin in IR were confirmed in a rat model of cerebral [75], renal [76] and pulmonary [89] IR. In these studies, the anti-inflammatory effects of hirudin, including a reduction in leukocyte adhesion, was systematically observed [90].

2. Melagatran

In a porcine model of kidney transplantation, direct inhibition of thrombin via melagatran improved kidney function and decreased inflammatory response and histological lesions one week after transplantation [78]. This beneficial effect was confirmed in the same model up to 3 months after transplantation [91] in concert with a reduction in fibrosis [92]. A previous study did not find any protective effects of melagatran on renal reperfusion injury [77]. The shorter ischemia duration in this study is probably the origin of this discrepancy in results. Melagatran was withdrawn from the market in 2006 due to the risk of liver toxicity.

3. Dabigatran

Initially, dabigatran’s effects on cerebral IR were studied in a model of transient occlusion of the middle cerebral artery in mice and rats [79,80]. In these models, dabigatran had no beneficial effect on the area of necrosis but reduced the risk of hemorrhagic transformation in comparison with warfarin. The neurovascular protective effect of dabigatran was implied by these studies. As with rivaroxaban, Dittmeier et al. found a reduction of infarct size without increasing the rate of intracerebral hemorrhages in a model of transient occlusion of the middle cerebral artery [71]. Although dabigatran had a beneficial effect in a renal IR model [60], no cardioprotective effect was observed in a model of renal IR in rats [81]. More recently, dabigatran treatment improved vascular integrity via sinusoidal protection in a model of hepatic IR [82].

#### 3.2.3. Inhibitors of the TF/FVIIa Complex

Apart from activation with thrombin, PAR-1 and -2 can also be activated via the complex formed by FT and FVIIa. One of the ways to inhibit this complex is to use a competitive inhibitor of FVIIa: active site-inhibited FVIIa (ASIS) or even a monoclonal antibody directed against TF. Inhibition of the FVIIa-TF complex via the ASIS molecule reduced myocardial reperfusion injury in mice [84]. In rabbits, in a model of myocardial IR, the use of a similar recombinant molecule made it possible to reduce not only the size of infarcts but also the no-reflow phenomenon [83]. In rats, inhibition of FVIIa decreased vascular permeability [65]. In a rabbit model of myocardial IR, administration of a monoclonal antibody directed against TF before the start of reperfusion significantly reduced the final size of the infarct as well as leukocyte infiltration [74].

When all studies performed using different AC therapies and models are analyzed, it seems that these therapies are protective against IR injury lesions. The discrepancies between studies are reliable in terms of species and protocol of administration used. It should be emphasized that tissue specificity must be considered. Indeed, when all models of cerebral IR are considered, AC therapy, via neurovascular protection, has beneficial effects in decreasing hemorrhagic complications, while it tends to decrease infarct size in myocardial infarction models. The beneficial effects of anticoagulant therapy are therefore organ specific.

## 4. Anticoagulant Therapies in the Field of Transplantation

Numerous strategies have been developed to prevent or treat IR injuries. The literature abounds with various types of interventions, making it impractical to enumerate all of them comprehensively. However, we can mention remote conditioning as an example; it is a very safe intervention, though its relevance is questionable, as current clinical studies have yielded negative results. Targeting the mitochondria is an interesting therapeutic approach. Recent advancements include the successful application of a spermidine inhibitor and the infusion of cyclosporine just prior to vessel clamping. These therapeutic strategies are promising, but their safety has not yet been extensively tested for organs other than the kidneys. Interventions focusing on innate immunity, especially those inhibiting the complement system, deserve special mention for their promising outcomes in non-human primate models of donation after brain death.

AC therapy, already a staple in clinical practice, offers a significant opportunity in transplantation, as these therapies might improve IR-induced tissue injury. It is therefore of interest to use them in the context of transplantation, particularly to improve graft during storage and/or preconditioning. Optimizing their benefits necessitates a precise understanding of therapeutic targets and identifying the best timing and methods for infusion. Unfortunately, very few studies have been performed in this context, particularly considering potential hemorrhagic risk (see below). Most of the studies concern kidney transplantation. A recognized model of kidney transplantation in pigs allowed for the testing of the impact of graft conservation on early and delayed graft outcome. Initially, melagatran used before ischemia and/or during graft conservation prevented delayed graft function [78] and kidney fibrosis 3 months later [92]. It is noticeable that melagatran added only to preservation solutions improved graft outcome. No hemorrhagic complications were observed during surgery or over the days following transplantation. Since then, several molecules have been tested in the same model, including fondaparinux [68] and a bispecific ACthat inhibited both thrombin and FXa (EP217609) [85]. The same benefits were observed, without increased hemorrhagic risk, when ACs were added to the preservation solution. In practice and despite the very few studies, it might be suggested that Acs, via their anti-inflammatory effect and protection of endothelium integrity, could be of interest in conservation solutions. 

Today, hemorrhagic risk to both the donor and the recipient, which haunts surgeons and anesthetists, is a limitation for using ACs in the context of transplantation. Thus, ensuring their safety while maintaining efficacy is crucial, requiring evaluations that are as thorough in assessing safety as they are in confirming effectiveness. Considering this hemorrhagic risk, it is not acceptable that AC therapy be administered during the reperfusion period after organs have been transplanted. Development of new ACs and more precisely anti-FXI and/or -FXII, whose hemorrhagic risk is lower than current Acs, could be beneficial not only in organ conservation but also during surgery and particularly after reperfusion (see Section 5).

### 4.1. Risk of Bleeding

A clinician’s apprehension, whether substantiated or not, regarding the utilization of new AC therapy during invasive procedures is rooted in the consideration of the bleeding risk. Certainly, the advantages of AC therapy are consistently outweighed by the potential for significant bleeding events. This is precisely why all novel therapies in this domain are accompanied by safety data focused on this very point. The consideration of this matter necessitates the incorporation of four pivotal concerns: the anticipated benefits, the procedural bleeding risk, the pharmaceutical bleeding risk and the patient’s susceptibility to bleeding. As we have explored the advantages of novel AC drugs in the preceding chapters, in this section, our attention now shifts to examining the bleeding risk associated with the patient (deceased organ donor), the multi-organ procurement procedure and the pharmaceutical-related bleeding risk.

#### 4.1.1. Association with the Multi-Organ Procurement Procedure (MOPP)

Quantifying the bleeding risk inherent in a MOPP remains a formidable challenge, primarily attributable to the lack of comprehensive data in this domain. While isolated case reports have been disseminated, the absence of dedicated observational studies further compounds this issue. Consequently, we are compelled to extrapolate the potential bleeding risk during MOPP by drawing upon insights gleaned from the assessment of risks associated with significant surgical interventions. In theory, MOPP is a procedure fraught with inherent risks, owing to its requisite creation of a sizable surgical incision for optimal organ exposure coupled with the intricate dissection of extensive vascular networks. Stratifying the bleeding risk for a particular organ is a challenging endeavor and is probably impossible. 

Typically, the assessment of the bleeding risk associated with a specific invasive procedure results from the interplay between the patient’s individual propensity for bleeding and the inherent bleeding risk associated with the procedure itself [93,94,95,96]. 

In the field of organ donation, we encounter two patient categories: those who become donors following brain death and those who pass away due to circulatory death, which can be uncontrolled (uDCDD) or controlled (cDCDD). In this final category, our intentional focus is directed towards cDCDD, as it stands as the predominant reason for organ donation after circulatory death. Both DBDD and cDCDD share commonalities in the form of catastrophic brain injury and the side effects of intensive care unit (ICU) admission. The influence of brain injuries on coagulation is well recognized, with comprehensive data available for traumatic brain injury (TBI) and strokes (which account for 25% and 40% of brain death cases, respectively). In the case of TBIs, there is a decline in platelet count and function from ICU admission, which subsequently stabilizes or even increases beyond admission levels within 3 days. Conversely, the coagulation cascade and fibrinolysis undergo a surge within the first day post injury then returning the normalcy [97]. vWF appears to assume a pivotal role in these alterations [98]. These hemostatic disturbances shift manifest in two-thirds of all cases [97]. In the event of a stroke, platelets become highly activated and are rapidly consumed. At the same time, we can observe an increase in coagulation cascade activation. Similarly, in cases of cardiac embolism etiology, fibrinolysis is activated [99]. Documentation regarding the influence of the ICU on hemostasis and inflammation remains limited, possibly due to the diverse array of situations encountered. For instance, when considering TBI, there is notable prevalence of concurrent trauma, often requiring extensive blood product transfusions as part of treatment. Moreover, it is noteworthy that thrombo-embolism stands out as a prevalent side effect among the ICU patient population. 

Nonetheless, the disparities between DBDD and cDCDD arise from variations in factors such as the length of ICU stay, the distinct physiopathology underlying brain death and the intricate procedural approach to cDCDD, particularly in instances involving normothermic regional perfusion (NRP) [6].

For instance, in major non-cardiac surgeries, the occurrence of hypotension resulting from blood loss ranges from 9% to 14% [100]. An alternative approach for assessing perioperative bleeding during a MOPP involves the utilization of blood products, including red cell units and other derived components. Within a French multicentric DBDD cohort study, it was observed that approximately 15 to 30% of procedures necessitated the administration of red blood cell units, plasma and platelets to manage per-operative anemia [101]. This approach lacks precision, as transfusion of red blood units primarily addresses anemia to enhance oxygen transport and does not serve as a distinct indicator of significant bleeding events.

#### 4.1.2. Association with Anticoagulant Agents

For invasive procedures, numerous guidelines mandate the cessation of all anticoagulant therapies for several days beforehand [102,103].

The perioperative administration of anticoagulants during elective surgery is frequently deemed essential due to the implantation of specific medical devices (such as cardiopulmonary bypass machines, intravascular stents, percutaneous valves, rhythmologic procedures, etc.) as well as vascular clamping. The leading molecule used is the UFH, associated or not with anti-aggregants [104]. Despite a notably limited level of evidence, decades of using this molecule have established it as the benchmark AC in both experimental and clinical studies. Fondaparinux appears to have a safety profile that is at least equivalent to those of UFH and LMWH [105]. The safety profile of the anticoagulant agent was explored specifically within the limited scope of medical complications, such as spontaneous bleeding, particularly of the intracranial type. There is a lack of evidence in a surgical context, and the available data are scant, highlighting only very specific acquired coagulopathies [106]. Consequently, the use of alternative and efficient anticoagulant agents needs to be guided by pharmacokinetic and pharmacodynamic (PK/PD) concerns.

### 4.2. Normothermic In Situ and Ex Vivo Reconditioning

The use of extended-criteria donor grafts for transplantation has necessitated the rise of normothermic perfusion reconditioning. There are two main strategies: in vivo NRP [107] or normothermic ex vivo perfusion [108]. Both methods rely on extracorporeal circulation devices, which require AC therapy. Both techniques require AC therapy using UFH. This assertion stems from a limited understanding of alternative AC options.

Even though this stage in the transplantation pathway of the graft appears promising in theory, we lack the robust preclinical or clinical data to substantiate this perspective.

### 4.3. Optimizing Outcomes: A Strategic Guide to Implementing AC Therapies during Transplantation Procedures

There is a dual challenge for care providers in determining the optimal time to administer AC therapy for maximum benefit while also considering bleeding risk. To aid in decision-making, we have guidelines, but they lack detailed information on this practical issue. Effective communication between the surgical team and other care providers, in concert with a personalized treatment approach, assessing the balance between bleeding and thrombosis through point-of-care tests such as thrombo-elastometry, for instance, seems to be of major importance. Unfortunately, there are limited data to support this approach.

In this section, we present a practical approach to using AC therapies for graft optimization.

We could divide the graft transplantation process in four distinct steps:

1. Deceased donor management
2. Multi organs harvesting
3. Preservation
4. Reperfusion in recipient

**Figure 2:**
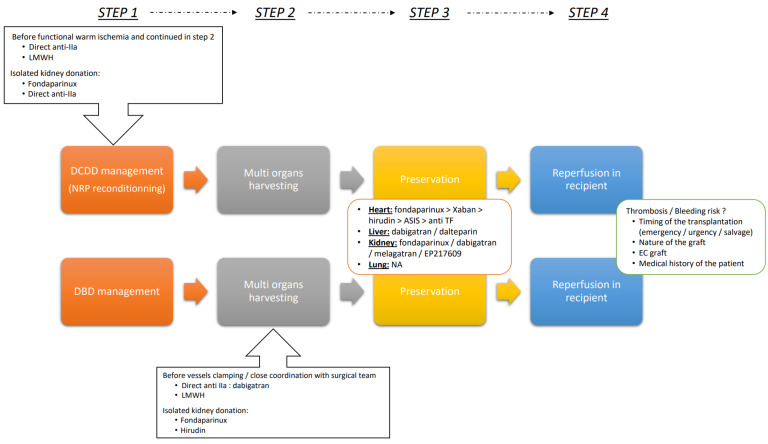
Proposal for a strategic guide to implementing AC therapies during transplantation procedures. STEP 1 focuses on the nature of the deceased donor (DCDD or DBD), emphasizing the role of AC therapy and the careful selection of molecules and safety measures for all organs involved. STEP 2 relates to multi-organ harvesting in terms of either continuing the same AC therapy in DCDD with NRP or initiating it in DBD cases, with coordination with the surgical team being critical for optimal timing. STEP 3 explores AC therapy preconditioning, varying molecules and therapy duration and incorporating it into preservation solutions, supported by experimental studies and the new avenue of ex vivo normothermic perfusion. STEP 4 considers graft reperfusion in the recipient, which demands careful consideration of safety, balancing risks between bleeding and thrombosis, while factoring in the patient’s medical history and the specific organ graft involved. AC: anticoagulant therapy; ASIS: active site-inhibited FVIIa; DBD: donor after brain determination of death; DCDD: donor after circulatory determination of death; EP217609: anti-Xa and anti-IIa activity; NA: not applicable; NRP: normothermic regional perfusion; LMWH: low molecular-weight heparin.

For using AC therapy to optimize the graft, caregivers need to assess the balance between the beneficial effects and the risks associated with each of these steps, as illustrated in Figure 2.

In the initial step of deceased donor management, the nature of the deceased donor–whether DCDD or DBDD, is a crucial consideration. While AC therapy seems less relevant to DBDD cases, it offers a pivotal improvement for DCDD. In these donors, AC therapy (specifically UFH) is already mandatory prior to the onset of functional warm ischemia. Refining the choice of molecule and target within the coagulation pathway seems pertinent. Another important consideration during this step is ensuring the safety of conditioning for all organs involved. Some AC therapies do not affect certain organs, as detailed in Table 1. Hence, it is crucial to choose a therapy that offers protection to all selected organs for harvesting. 

In the second step, during multi-organ harvesting, two options must be considered. First, if it is a DCDD with NRP, it is imperative to continue using the same molecule chosen in the initial step. Second, if it is a DBDD, this is the optimal time to administer the AC therapy agent. Close coordination with the surgical team is vital, as it is essential to determine the ideal moment to infuse the treatment, which is typically after the major dissection and before clamping the vessels.

The third step emerges as a promising phase for AC therapy preconditioning. The choices of molecules and the duration of AC therapy can be varied. ACs can be incorporated into the preservation solution, whether for static cold storage or hypothermic machine perfusion. Preliminary data from experimental studies supports this. Ex vivo normothermic perfusion presents a fresh avenue for evaluating graft function, and these devices require AC therapy for operation.

The final step, graft reperfusion in the recipient, demands meticulous attention to safety. Factors to consider include the organ’s vulnerability, a balance between risks of bleeding and thrombosis—which hinge on the patient’s medical history—and the graft (e.g., heart, lung, kidney, liver) also plays a critical role in this evaluation.

## 5. Perspectives

As previously described, ACs, via their anti-inflammatory effects, could be of interest in the context of transplantation either in preserving graft and preventing IR injury after transplantation. Unfortunately, most of the ACs tested need supra-therapeutic concentrations and thus bear a real hemorrhagic risk. Apart from the context of IR injury, a number of molecules were developed either as anti-coagulation factors or to modulate PAR’s signaling.

### 5.1. Targeting Contact Phase

Targeting contact phase could be a very attractive perspective in transplantation-induced thrombo-inflammation. Various arguments exist suggesting a clinical interest of contact phase inhibitors in organ transplantation. Indeed, during the preservation of the organ on perfusion machines, the passage of blood through tubules activates coagulation via the contact phase [109] by adsorbing to artificial surfaces and activating FXII. This activation of contact phase can lead to thrombus formation, intervene in innate immunity [110] and activate the complement system [111] and platelets [112]. Different molecules targeting this phase have already been tested in the prevention of thrombosis [113]. Inhibition of FXIIa via a humanized monoclonal antibody (IgG 3F7) has prevented thrombosis in an extracorporeal membrane oxygenation (ECMO) rabbit model [114] without deleterious effects on hemostasis. We can easily imagine that the use of anti-FXIIa could be of interest in the conservation phase of harvested organs, particularly when using perfusion devices.

Another phase in the transplantation process requiring an anti-inflammatory action is when the organ is transplanted. At this time thrombo-inflammation induced-reperfusion injuries could be modulated by inhibiting contact phase without increasing bleeding events. Indeed, FXII activation could lead to endothelium dysfunction with increase vascular permeability [115]. Thus inhibiting this pathway during or just after transplantation could decrease thrombo-inflammation and ameliorate graft function without bleeds.

### 5.2. Targeting PAR Signaling

As described in a previous chapter, PAR signaling plays an important role in the deleterious effect of IR injuries. Thus, targeting these receptors is of interest. For many years, orthosteric drugs directing PAR-1, such as vorapaxar, have been developed and tested in clinical conditions such as acute coronary syndrome (TRACER study) [116]. Although it reduces cardiovascular or ischemic events, vorapaxar has been associated with an increased risk of major bleeding. Under these conditions, allosteric drugs directed against PARs, such as parmodulins, have been developed to prevent side effects such as bleeding events. Parmodulins are small chemical compounds that bind to the intracellular face of PARs [117]. ML161 is a well-studied parmodulin which reduces the expression of tissue factor in TNFα-activated endothelial cells and decreases platelet aggregation [118]. In a mouse model of myocardial infarction [119], IV injection of 5 mg/kg of ML161 30 min before ischemia reduced infarct size via inflammasome suppression and had cytoprotective effects [119].

Considering hemorrhagic risk in the context of organ transplantation, development of new molecules that target contact phase of coagulation or modulate PARs signaling should be of interest particularly in the phase of organ reperfusion in reducing thrombo-inflammation phenomenon and tissue injury. These “modulations” could ameliorate early and delayed graft outcomes particularly when using organs from deceased without increasing bleeding events. Considering the safety of such treatment, clinical trials on humans should be performed.

## 6. Patents

Notice n° WO2012146774 dépôt le 1 November 2012 et Notice n° FR2974478 dépôt le 28 April 2011.

Conjugués d’Oligosaccharide dans la Prévention de l’Ischémie-Reperfusion.

Classification CIB: A01N 1/02; C07H 15/26.

Classification CPC: C07H 15/08; A01N 1/0226; C07H 15/26.

Famille de brevets: WO2012146774A1; FR2974478A1.

## Figures and Tables

**Table 1:** Effects of anticoagulant therapies in different animal ischemia–reperfusion models.

		Species	Models	In Vivo Effect	Reference
UFH		Rabbit	Global MI	Decreases creatine kinase, improves left ventricular end-diastolic pressure	Friedrich, 1994 [49]
		Dog	MI	Reduces the extent of myocardial injury	Black, 1995 [50]
		Dog	MI	Decreases infarct size	Libersan, 1998 [51]
		Rat	Cerebral IR	Reduces infarct size	Yanaka, 1996 [56]
		Pig	Liver IR	Decreases hepatic enzymes compared to control	Liu, 1999 [53]
		Rabbit	Hepatic IR	Improves hepatic IR injury caused by sinusoidal microcirculatory disturbance	Matsumoto, 2000 [54]
		Rat	Intestinal IR	No effect on inflammatory response	Schoots, 2004 [55]
LMWH	Enoxaparin	Dog	MI	Decreases infarct size	Gralinski, 1996 [57]
		Dog	MI	Decreases infarct size	Libersan, 1998 [51]
		Dog	MI	Decreases infarct size	Thourani, 2000 [58]
		Rat	Mesenteric IR	Improves hemodynamics parameters during reperfusion	Walensi, 2013 [59]
		Rat	Kidney IR	Decreases prolidase and malondialdehyde levels	Yazici, 2016 [60]
	Dalteparin	Rat	Liver IR	Prevents increase of serum transaminases and increases hepatic tissue blood flow	Harada, 2006 [61]
	ULMWH	Rat	Cerebral IR	Decreases infarct size and increase neurological deficit scores	Zhang, 2007 [62]
	Reviparin	Rat	Kidney transplantation	Decreases albuminemia and cellular infiltration	Gottman, 2007 [63]
Fondaparinux		Rat	Kidney IR	Increases survival and decrease creatinine levels	Frank, 2005 [64]
		Rat	Intestinal IR	No effect	Olenders, 2005 [65]
		Rat	Heart IR	Improves post ischemic myocardial contractile performance and tissue damage	Montaigne, 2008 [66]
		Rat	MI	Decreases infarct size	Macchi, 2014 [67]
		Pig	Kidney transplantation	Improves graft outcome in both the acute and chronic phases	Tillet, 2015 [68]
Direct anti-Xa	Rivaroxaban	Mouse	Mesenteric IR	Attenuates the leukocyte–platelet–endothelial interaction	Iba, 2014 [69]
		Rat	Cerebral IR	No effect on infarct size but improves paraparesis score	Kono, 2014 [70]
		Rat	Cerebral IR	Develops significantly smaller strokes and less severe functional deficits	Dittmeier, 2016 [71]
		Rat	Cerebral IR	No effect on infarct size	Morihara, 2017 [72]
		Rat	MI	Decreases infarct size	Guillou, 2020 [73]
	Apixaban	Rat	Cerebral IR	No effect on infarct size	Kono, 2014 [70]
Direct anti-IIa	Hirudin	Rabbit	MI	Decreases infarct size	Erlich, 2000 [74]
		Rat	Cerebral IR	Decreases infarct size	Karabiyikoglu, 2004 [75]
		Mouse	Bilateral kidney IR	Protects from renal failure	Sevastos, 2007 [76]
	Melagatran	Rat	Kidney IR	No effect	Nitescu, 2007 [77]
		Pig	Kidney transplantation	Improves graft outcome and reduces renal injury	Giraud, 2009 [78]
	Dabigatran	Mouse	Cerebral IR	Does not increase hemorrhagic risk after thrombolysis	Sun, 2013 [79]
		Rat	Cerebral IR	Reduces hemorrhagic complication after thrombolysis	Kono, 2014 [80]
		Rat	Cerebral IR	Reduces infarct size without an increase in intracerebral hemorrhage	Dittmeier, 2016 [71]
		Mouse	MI	No effect on infarct size	Hale, 2015 [81]
		Rat	Kidney IR	Decreases prolidase and malondialdehyde levels	Yazici, 2016 [60]
		Mouse	Liver IR	Prevents increase of serum transaminases and improves vascular integrity	Noguchi, 2021 [82]
Other ACs	ASIS	Rabbit	MI	Decreases infarct size	Golino, 2000 [83]
		Rat	Intestinal IR	Improves endothelial permeability in the ileum	Olanders, 2005 [65]
		Mouse	MI	Decreases infarct size	Loubele, 2009 [84]
	Anti-TF (MoAbs)	Rabbit	MI	Decreases infarct size	Erlich, 2000 [74]
	EP217609	Pig	Kidney transplantation	Improves graft outcome and reduces renal injury	Tillet, 2016 [85]

AC: anticoagulant; ASIS: active site-inhibited FVIIa; EP217609: anti-FXa and anti-FIIa activity; IR: ischemia–reperfusion; LMWH: low molecular-weight heparin; MI: myocardial infarction; MoAbs: monoclonal antibodies UFH: unfractionated heparin; ULMWH: ultra-low molecular-weight heparin; TF: tissue factor.

## Data Availability

Not applicable.
